# Nanohardness and Young’s Modulus of Pb_1–*x*_Cd_*x*_Te Crystals Grown by the SSVG and MBE Methods

**DOI:** 10.1021/acsomega.3c06502

**Published:** 2023-11-21

**Authors:** Wojciech Wołkanowicz, Stanisław Adamiak, Anna Juś, Elżbieta Łusakowska, Roman Minikayev, Krzysztof Dybko, Dariusz Płoch, Jędrzej Korczak, Andrzej Szczerbakow, Tomasz Wojtowicz, Wojciech Szuszkiewicz

**Affiliations:** † Institute of Physics, Polish Academy of Sciences, al. Lotników 32/46, 02-668 Warsaw, Poland; ‡ Institute of Materials Engineering, University of Rzeszow, Pigonia 1, 35-959 Rzeszow, Poland; § International Research Centre MagTop, Institute of Physics, Polish Academy of Sciences, al. Lotników 32/46, 02-668 Warsaw, Poland

## Abstract

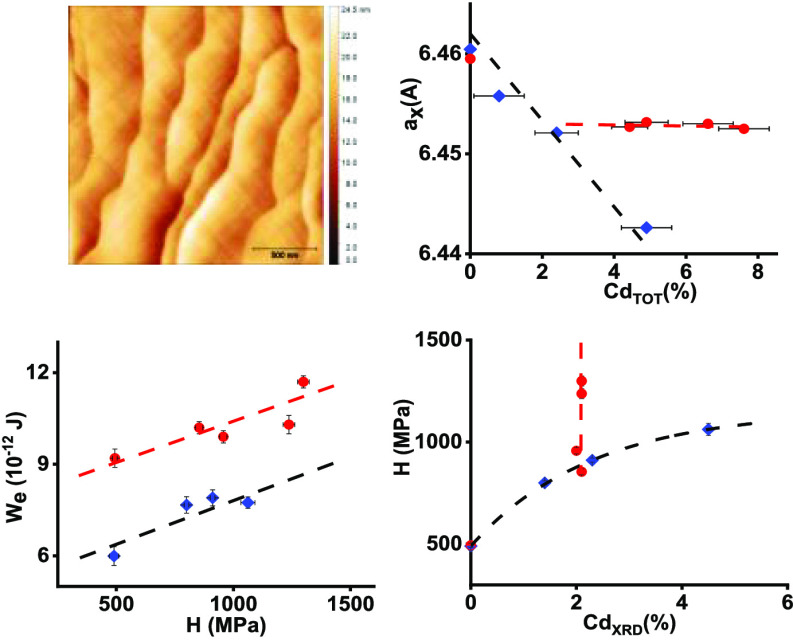

The nanohardness and Young’s modulus of Pb_1–*x*_Cd_*x*_Te single crystals prepared by the self-selecting vapor growth (SSVG) method and thick, MBE-grown layers with a total Cd content of up to 7% metal atoms were studied using the nanoindentation technique; the nanohardness and Young’s modulus were calculated by the Oliver and Pharr method. Significant hardening of SSVG crystals with increasing number of Cd atoms replacing Pb atoms in the formed solid solution was observed, and low anisotropy of the nanohardness and Young’s modulus were found. The CdTe solubility limit in the solid solution grown using an MBE equal to 2.1% was demonstrated; even for the significantly higher total Cd concentration in the layer, the possible presence of precipitates was not detected. Significant differences were found for both the energy of elastic crystal deformation and Young’s modulus determined for samples grown using the two methods. An increase in nanohardness with an increase in the number of Cd atoms outside the cation sublattice was shown. The different ratios of hardening mechanisms acting simultaneously in the analyzed crystals in various ranges of Cd concentrations were demonstrated and discussed. The observed effects were attributed to the much higher concentration of point defects in MBE-grown layers than in SSVG crystals, in particular, the interstitial Cd–Te vacancy complexes effectively hampering nucleation and propagation of dislocations in the former case.

## Introduction and Motivation

Group IV–VI semiconducting compounds, in particular, lead-based chalcogenides as well as solid solutions based on them, have been extensively investigated for several decades both for their interesting physical properties and their attractive applications, mainly as thermoelectric devices and mid-infrared detectors.^1^ From the end of the last century, together with the development of low-dimensional technologies, new possible applications, such as elements for infrared optics, have also appeared. PbTe is probably the most studied and best-known representative of this group of materials. Both undoped PbTe or PbTe as a constituent of several systems (solid solutions, low-dimensional structures, composites, etc.) have attracted increasing interest in the last dozen years. Numerous reasons for this include new physical findings, for example, crystalline topological insulators and their properties,^2−4^ dynamic local symmetry breaking,^5,6^ a significant modification of the lattice dynamics,^7−9^ and possible wider applications of PbTe-based materials in thermoelectric energy conversion systems operating in the intermediate temperature range (500–850 K).^10^

The conversion of heat to electricity by thermoelectric devices may play a key role in energy production and utilization. Materials for thermoelectric generators or refrigerators should exhibit high thermoelectric performance as well as mechanical stability. The improvement of mechanical properties is clearly desirable in the production process and mass application of devices. Apart from the extensive search for materials with a high thermoelectric figure of merit, zT, desired for thermoelectric devices,^11−17^ present studies are also focused on their mechanical properties, such as hardness, elastic properties, and brittleness.^14−19^ The hardness and Young’s modulus are frequent topics of studies reported in the last years. Hardness is a measure of the resistance of a material to plastic deformation, and Young’s modulus determines the stiffness of a material.

Crystals containing PbTe and CdTe, studied for many years, continue to be subjects of active research today. The phase diagram of this system demonstrated strong temperature-dependent CdTe solubility in PbTe.^20−22^ The low solubility results from the differences in the crystal structure of these compounds. PbTe crystallizes in the *fcc* rock-salt-type structure (space group *Fm*3̅*m*), whereas CdTe crystallizes in the zinc blende-type structure. Despite the almost perfect lattice parameter match between PbTe (6.462 Å) and CdTe (6.481 Å), the phase diagram does not permit the growth of bulk crystals containing more than 2% CdTe from the melt by routine growth methods.^23^ The successful growth of single Pb_1–*x*_Cd_*x*_Te crystals by the self-selecting vapor growth (SSVG) method changed this situation.^24−26^ Several properties of solid solutions in a much wider composition range than those studied previously, including structural and elastic properties, modification of the PbTe band structure, or electron and heat transport properties, are known today.^25−31^

The aim of the present study was to determine the dependence of selected mechanical properties, the nanohardness (*H*), and Young’s modulus, (E), on the Cd content in Pb_1–*x*_Cd_*x*_Te crystals obtained using two growth techniques. The depth-sensing nanoindentation technique is a widely contemporarily used method for the characterization of these properties and was selected for our investigations. At least one natural (100)-oriented face was present in bulk crystals; therefore, the same orientation of MBE-grown layers, resulting from the proper choice of substrate, was selected to compare the properties of the two materials. The same experimental setup was also used for the measurements. Recently, it was demonstrated that the nanohardness of PbTe bulk crystals and MBE-grown layers differ noticeably.^32^ On the other hand, analogous differences are not observed in some semiconducting compounds, for example Cd_1–*x*_Hg_*x*_Te.^33^ Therefore, an interesting question arises as to whether this effect also exists in Pb_1–*x*_Cd_*x*_Te crystals.

## Samples and Experimental Details

All investigated bulk crystals were grown by the SSVG method.^25,26^ For the PbTe growth, the synthesis of elemental Pb and Te of 5N purity from Alfa Aesar Company was performed first in the presence of an excess of about 1% Te. After the synthesis, an excess of Te was removed, and the obtained compound served as a starting material for the SSVG process.^32^ The Pb_1–*x*_Cd_*x*_Te samples were grown from polycrystalline PbTe and CdTe and synthesized with an excess of Te and Cd, respectively.^26^ These materials were loaded into quartz ampules; the crystal growth by an evaporation–condensation process was carried out at temperatures below the PbTe melting point of 924 °C^22^ and typically ranged from about 810 to 870 °C.^26,30^ Finally, the crystals grown at high temperatures were quenched to room temperature. The obtained faceted, single crystals exhibited high structural perfection. Their volumes varied from a few hundred cubic millimeters to more than one cubic centimeter.

Thick Pb_1–*x*_Cd_*x*_Te layers with various Cd contents were grown on a commercially available, epi-ready GaAs substrate by MBE using two systems successively. First, the (100)-oriented or slightly misoriented (of the order of one degree) GaAs wafers (from AXT Company) were overgrown with 4 μm thick CdTe buffer layers using the EPI 620 system. The dependence of the crystallographic structure of the MBE-grown layers on the misorientation of GaAs substrate is a well-known phenomenon, and it was observed previously not only for a number of II–VI semiconducting compounds and their solid solutions but also for many other materials (e.g., zinc blende MnTe^34^). Between the buffer and substrate, a thin ZnTe layer (about 7 nm thick) was deposited to achieve growth along the [100] direction. Before layer growth, the substrate prepared in such a manner was etched in HCl to remove oxides and impurities. Next, the growth was performed at 270 °C using another MBE system equipped with effusion cells of elemental Pb, Cd, and Te. Just before the epilayer growth, a thin CdTe film was deposited to obtain a perfectly smooth surface. The whole process was monitored in situ by reflection high-energy electron diffraction (RHEED) to confirm the epitaxial growth mode and to verify the crystal quality of the obtained layers.

The crystal structure of Pb_1–*x*_Cd_*x*_Te grown by the SSVG method was determined by powder X-ray diffraction (XRD) utilizing a Philips X’Pert (PANalytical) diffractometer and Cu Kα_1_ radiation. The diffraction spectra for 2θ values ranging from 20 to 150° were accumulated, and the Rietveld refinement software fullprof.2k (v.7)^35^ allowed the precise determination of the crystal lattice parameter *a*_*x*_ of the formed solid solution. The same diffractometer was used for studies of MBE-grown Pb_1–*x*_Cd_*x*_Te layers; in this case, the 2θ–θ experimental mode was used. The GaAs 400 Bragg peak served as an internal standard to determine the exact angular positions of the Bragg peaks for Pb_1–*x*_Cd_*x*_Te. The accuracy of the determined lattice parameter for all samples was equal to 10^–4^ Å.

The chemical composition of the solid solution was calculated from the lattice parameter *a*_*x*_ according to the formula
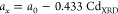
1where *a*_0_, the PbTe lattice parameter at room temperature, is 6.462 Å, and Cd_XRD_ is the CdTe content in the solid solution.^25^

SEM and energy-dispersive spectroscopy (EDS) measurements were performed using an Hitachi Flex 1000 scanning electron microscope with an accelerating voltage of 15 kV. No conductive coating was used as all samples were sufficiently electrically conductive. EDS was performed to determine the total Cd concentration in the investigated sample, that is the fraction of metal atoms in the crystal that were Cd atoms, Cd_TOT_. The accuracy of the CdTe content in solid solutions determined from XRD data was about 0.1%; however, the accuracy corresponding to the EDS method was not so high (about 0.7%). The surface morphology of the samples was studied using an atomic force microscope (AFM) (Innova–Bruker). The root mean square (RMS) parameter was applied for the surface roughness analysis, and its value was calculated over a 2 μm × 2 μm scan area. Electron transport measurements were performed for MBE-grown layers at room temperature using the Hall bars with In contacts, and the resulting free carrier concentration was estimated.

A depth-sensing Anton Paar Ultra Nanohardness Tester with a Berkovich-type diamond indenter tip was used for the nanoindentation. All nanoindentation measurements were performed at room temperature in air. Before planned measurements, the nanoindentation tester was calibrated using a fused silica standard. Each loading and unloading was performed in force control mode. The linear change of the load during application or removal of the load was equal to 33 μNs^–1^, and the time period of each maximum load was 15 s. Such a choice of experimental conditions reduced the influence of loading time and velocity and suppressed any tendency of layer cracking; a selected load period allows time-dependent plastic effects to diminish. The nanoindentation was performed at three different locations on the sample surface. Each set of measurements consisted of several independent indents (up to 25). Nanoindentations in one set of measurements were sufficiently spaced to prevent the possible appearance of mutual interactions. The average values of *H* and *E* were calculated for each set of data from the analysis of experimental curves using the method proposed by Oliver and Pharr.^36^

As at least one natural (001)-oriented face was present for all bulk crystals; 2 mm thick slices corresponding to this orientation were used for the nanoindentation measurements. In addition, 2 mm thick, (011)- and (111)-oriented (according to the Laue method) plates were carefully cut from the biggest-sized single Pb_1–*x*_Cd_*x*_Te crystal. These plates were mechanically polished and etched in a bromine–methanol solution to reduce the surface roughness and used for the determination of *H* and *E* anisotropy.

## Results and Discussion

**Figure 1:**
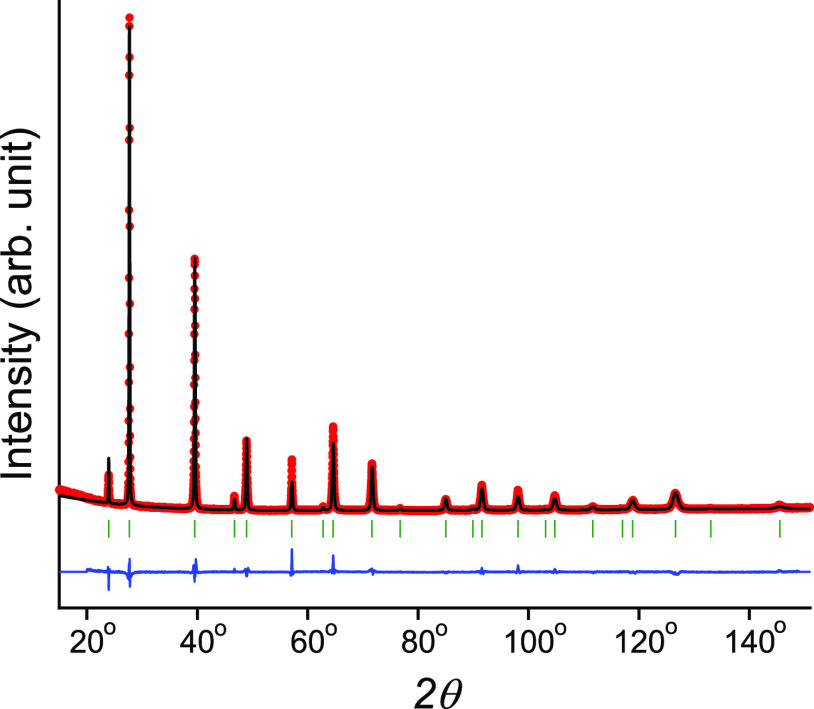
Rietveld refinement results for the Pb_1–*x*_Cd_*x*_Te sample #03 (see Table 1). The experimental points are indicated by dots (red), the calculated patterns by the solid line (black), and the difference (blue line) is displayed at the bottom. The positions of Bragg peaks are indicated as short vertical bars below the diffraction pattern.

**Figure 2:**
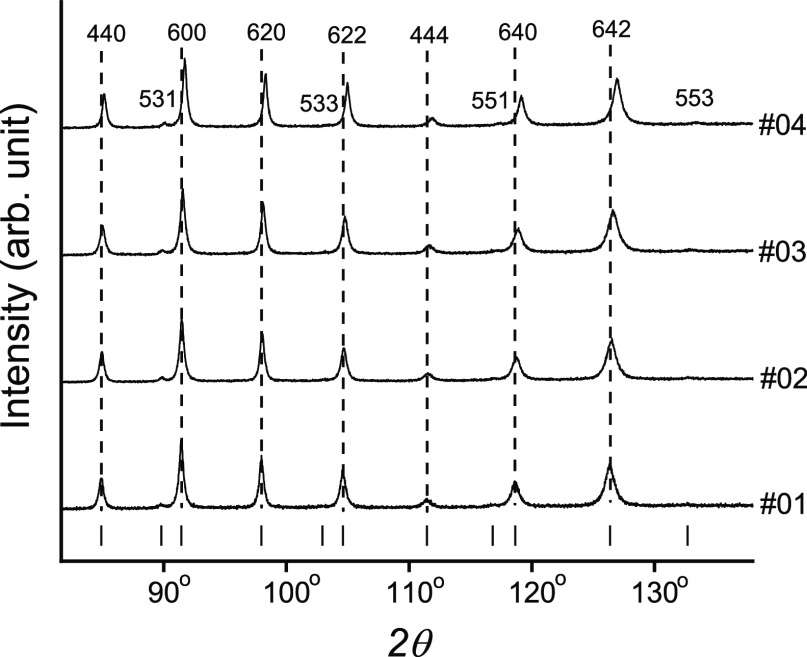
Comparison of diffraction patterns for bulk samples. Only a part of the results obtained for a high 2θ angle, highlighting a noticeable difference in the Pb_1–*x*_Cd_*x*_Te Bragg peak positions, is displayed. The positions of PbTe Bragg peaks are indicated as short vertical bars below patterns; dashed lines indicate the positions of principal peaks. Parameters of the samples in this and other figures can be found in Table 1.

The diffraction patterns of all SSVG crystals were obtained by powder XRD measurements and analyzed by Rietveld refinement. All diffraction peaks of Pb_1–*x*_Cd_*x*_Te can be well indexed to the face-centered cubic, rock-salt-type structure. No Bragg peaks corresponding to the possible presence of inclusions were observed. The experimental data were fitted with the calculated pattern, and both the intensity and form of peaks were perfectly reproduced. The result of a Rietveld refinement of Pb_1–*x*_Cd_*x*_Te crystals is shown in Figure 1 as an example. The refinement quality was the same for the other samples. Figure 2 presents a comparison of obtained powder XRD patterns for SSVG crystals. The observed composition-dependent shift of Bragg peak positions toward a higher angle confirms the decrease in *a*_*x*_ and the formation of a solid solution. To better demonstrate this effect, a part of the diffraction patterns only corresponding to the high index of Bragg peaks is shown in this figure. The linear intensity axis is used in Figures 1 and 2. The lattice parameter determined by Rietveld refinement and the Cd_XRD_ value resulting from it are given in Table 1. The obtained PbTe lattice parameter (the exact value 6.46046(8) Å) is in good agreement with previously reported 6.46179(3) Å,^37^ 6.46040(4) Å^38^ and 6.4616(3) Å.^39^ The Cd fraction of metal atoms in the crystal, Cd_TOT_, was independently calculated from EDS data and is also presented in Table 1. Within the limits of error, Cd_XRD_ and Cd_TOT_ values are equal for SSVG crystals. Therefore, during the growth of crystals by the SSVG method, Cd atoms introduced to the material replaced Pb atoms in the cation sublattice and formed the solid solution.

**Table 1:** Parameters of Samples Obtained by the Used Characterization Methods: the Lattice Parameter (*a*_*x*_), Cd Content (Cd_XRD_) Corresponding to *a*_*x*_, Cd Fraction of Metal Atoms (Cd_TOT_) Determined by EDS, Surface Roughness (RMS), and Layer Thickness (d)

**sample**	***d* [μm]**	***a**_**x**_***[Å]**	**Cd**_**XRD**_**(*x*)**	**Cd**_**TOT**_**(*x*)**	**RMS [nm]**
#01		6.4605	0	0	5.4
#02		6.4557	0.014	0.008	4.2
#03		6.4521	0.023	0.024	4.2
#04		6.4426	0.045	0.049	2.8
#05	2.1	6.4595	0	0	2.5
#06	1.4	6.4527	0.021	0.044	3.6
#07	2.3	6.4532	0.020	0.049	2.5
#08	2.3	6.4530	0.021	0.061	2.3
#09	2.2	6.4525	0.021	0.076	

Small nanoparticles CdTe-rich could be formed in Pb_1–*x*_Cd_*x*_Te with a temperature decrease.^40^ However, as-quenched typical ingot of Pb_1–*x*_Cd_*x*_Te containing 3% CdTe appeared to be single-phase alloy at 300 K within the resolution of SEM used for EDS measurements (∼50 nm).^23^ Pure PbTe, SnTe, and Pb_0.65_Sn_0.35_Te had a uniform crystalline structure and homogeneous compositions without any nanoscale inclusions, as directly demonstrated by transmission electron microscopy (TEM), whereas numerous inhomogeneities and nanostructures with a size distribution of 3–7 nm were observed in Na- and Bi-doped Pb_0.65_Sn_0.35_Te.^41^

The Cd content in all crystals was below the CdTe solubility limit, corresponding to the SSVG crystal growth at high temperatures,^22,21^ and crystal growth occurred under near-equilibrium conditions. The expected lack of precipitates in SSVG samples was confirmed by the results of XRD measurements. The literature data suggest that at least a residual fraction of Cd atoms forming point defects, such as interstitials, could be expected.^42,43^ Taking these facts into account, possible Cd aggregates or CdTe nanoprecipitates in SSVG crystals should also be excluded.

**Figure 3:**
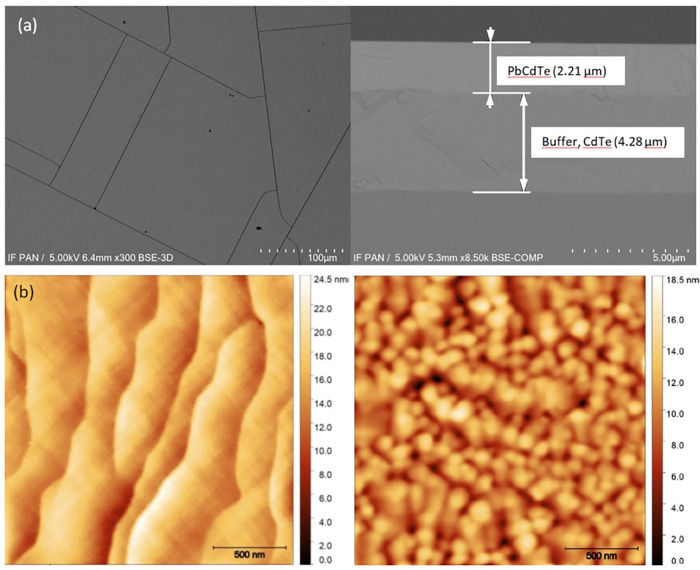
(a) SEM images showing cracks on the Pb_1–*x*_Cd_*x*_Te #09 sample surface resulting from different thermal expansion coefficients for the layer and buffer and the side view of this sample. (b) Surface morphology of the PbTe layer (sample #05) and Pb_1–*x*_Cd_*x*_Te layer (sample #08) by AFM (left and right, respectively).

SEM images demonstrated several cracks on the surface of MBE-grown layers due to thermal strain, resulting from a significant difference in the thermal expansion coefficient of PbTe and CdTe (α_PbTe_ = 20 × 10^–6^ K^–1^,^39,44,45^ α_CdTe_ = 4.7 × 10^–6^ K^–1^^46^). The mean distance between cracks was about 100 μm. Because of the thermal strain, numerous dislocations in the layers should be expected. The typical layer thicknesses determined by SEM were close to 2 μm. As an example, SEM images of the Pb_1–*x*_Cd_*x*_Te layer #09 are shown in Figure 3. The Cd_TOT_ of the MBE-grown layer determined by EDS, and its thickness are given in Table 1. This table also presents RMS values determined by the AFM method, indicating a similar surface roughness of a few nanometers for all samples.

The crystal structure of the layers was investigated by XRD using the same laboratory diffractometer as earlier. All Bragg peaks observed in the diffraction patterns corresponded to the (001) layer orientation. Apart from several peaks related to the layer, a few other peaks corresponding to the same orientation as the GaAs substrate and CdTe buffer were also found, and no possible trace of precipitates or secondary phases was detected. The angular position of the GaAs 400 Bragg peak served as an internal standard, and the lattice parameters determined for Pb_1–*x*_Cd_*x*_Te solid solutions are given in Table 1.

**Figure 4:**
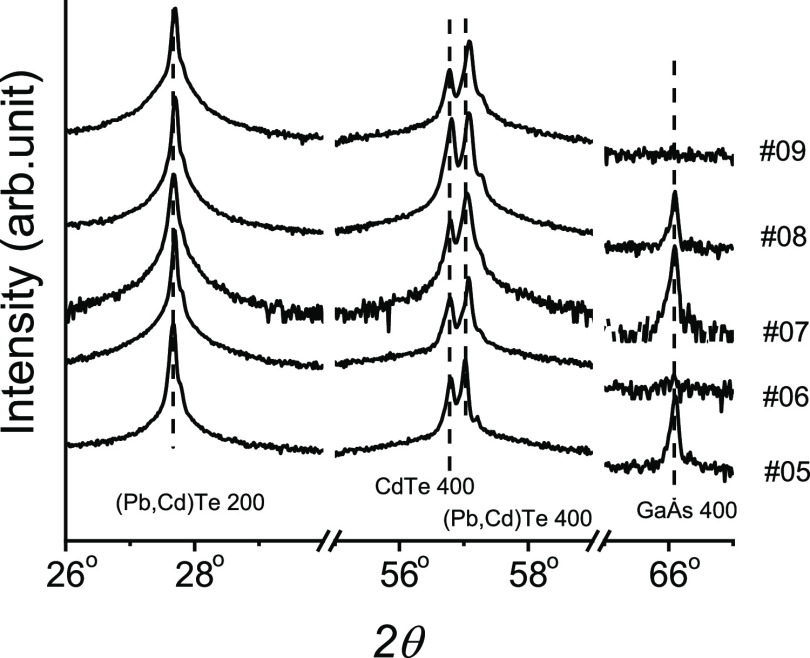
Comparison of diffraction patterns obtained for MBE-grown layers. The lowest pattern corresponds to PbTe.

**Figure 5:**
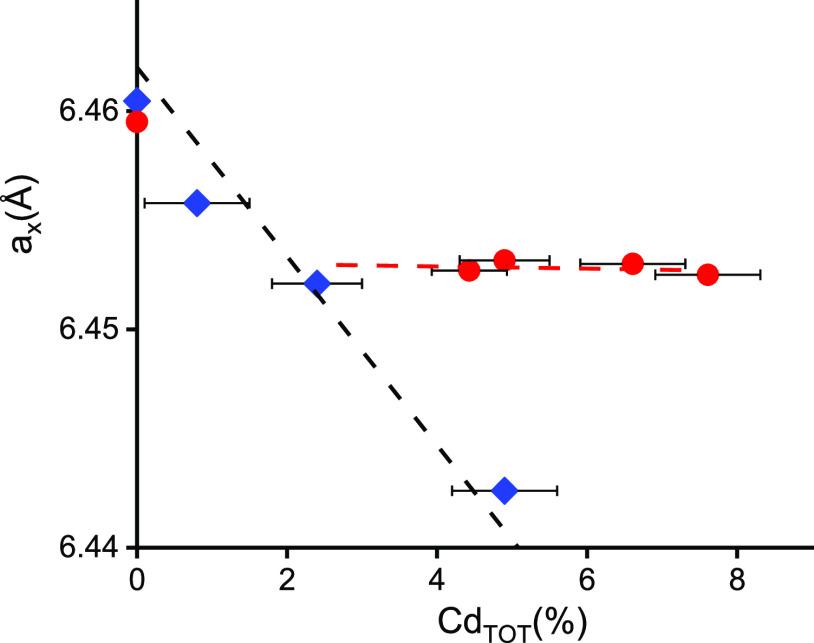
Lattice parameter of the formed solid solution (a_*x*_) versus the Cd fraction of metal atoms (Cd_TOT_) determined by EDS for bulk crystals (blue diamonds) and MBE-grown layers (red circles). The black dashed line was calculated using eq 1, assuming Cd_TOT_ = Cd_XRD_, and the horizontal red dashed line is the result of the linear fit.

A part of the obtained diffraction patterns is shown in Figure 4, and an identical angular position of the Pb_1−_*_x_*Cd_*x*_Te 400 Bragg peak for four layers with different high Cd_TOT_ values is observed. This finding indicates saturation of the lattice parameter and the same chemical composition of the formed solid solutions. The CdTe solubility limit in MBE-grown solid solutions equal to 2.1% was demonstrated. This effect is illustrated in Figure 5. Moreover, in spite of a huge (100% or more) difference in Cd_XRD_ and Cd_TOT_ values, a lack of detected precipitates or secondary phases indicates a very high concentration of Cd-related defects in these samples.

Electrical characterization revealed p-type conductivity for all crystals grown by the MBE and SSVG methods. The hole densities determined at room temperature in bulk crystals varied in a nonmonotonous manner between 1.2 × 10^18^ cm^–3^ for PbTe and 3.6 × 10^18^ cm^–3^ for Pb_1–*x*_Cd_*x*_Te according to the literature data.^26^ The p-type concentration determined at room temperature in this work for MBE-grown layers was noticeably lower than that for bulk crystals and was equal to (3.0 ± 0.5) × 10^17^ cm^–3^ for PbTe and (6.0 ± 0.5) × 10^17^ cm^–3^ for Pb_1–*x*_Cd_*x*_Te.

When studying the layer properties by nanoindentation, the result is dominated by these layer properties at low indentation depths. To avoid the possible influence of the substrate on the final result, the commonly accepted rule is to limit the nanoindentation depth to less than 10% of the layer thickness,^47^ a slightly higher value can also be found in the literature. The mechanical response is structure-dependent, but this effect is important for layer growth on a hard substrate, e.g., sapphire.^48^ After test measurements, the maximum load of 1 mN was selected for nanoindentation for SSVG-grown crystals and MBE-grown layers. Because of the use of a 4 μm thick CdTe buffer for Pb_1–*x*_Cd_*x*_Te layer growth, the maximum tip penetration depth from 150 to 250 nm seems to be the correct choice.

**Figure 6:**
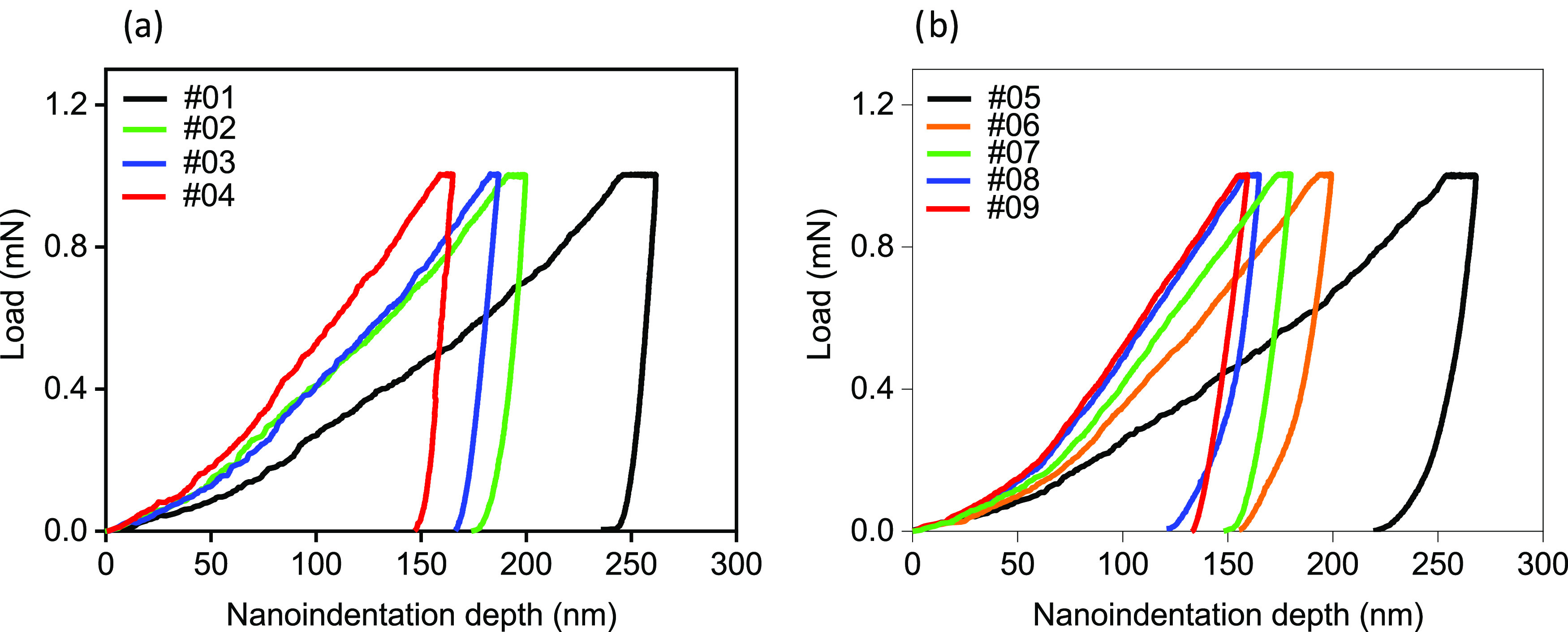
Nanoindentation curves obtained using a maximum load of 1 mN for bulk crystals (a) and MBE-grown layers (b).

The load–displacement curves determined by the nanoindentation for epilayers and the (001)-oriented natural face of bulk crystals are shown in Figure 6. Depth discontinuity, or the “pop in” effect, found for some other semiconductors and indicating initiation of the nucleation of both existing and newly created dislocations followed by their propagation within the crystal that occurs upon the onset of plastic deformation was not observed. It can be seen that the intender penetration increased under constant load for all crystals. This finding confirms that the maximum indentation depth is well above the depth associated with a transition from the elastic to an elasto-plastic regime. The attained sink-in distance of the order of several nanometers is composition-dependent; it corresponds to a creep from ∼6% for PbTe to about 3% for the hardest Pb_1–*x*_Cd_*x*_Te crystal. The total work energy, *W*_t_, required for a small material deformation is the sum of reversible (elastic) energy We and irreversible (plastic) energy *W*_p_. Values of these parameters, directly calculated from the nanoindentation curves are shown in Table 2; significant differences in *W*_e_ values determined for bulk crystals and MBE layers were found. The values of *H* and *E*, calculated according to the Oliver and Pharr method, are also given in this table. The comparison of *W*_e_ versus *H* dependence obtained for the two types of crystals is shown in Figure 8. A typical slow linear increase in *W*_e_ with increasing *H* is observed for both types of samples. The linear fit of this dependence was obtained for SSVG crystals using the following formula
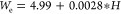
2and for MBE-grown layers by the following formula
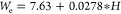
3which are also shown in Figure 8 by the dashed black and red lines, respectively. In order to find possible reasons of the observed differences, it is necessary to analyze the hardening mechanisms.

**Table 2:** Values of Parameters Determined by Nanoindentation^a^

**sample**	***W***_**e**_ **[10**^–**12**^ **J]**	***W***_**p**_ **[10**^–**12**^ **J]**	***W***_**t**_ **[10**^–**12**^ **J ]**	***H* [MPa]**	***E* [GPa]**
#01	6.0 ± 0.2	109.8 ± 2.4	115.8 ± 2.4	490 ± 20	57.5 ± 1.9
#02	7.7 ± 0.1	78.1 ± 0.6	85.8 ± 0.7	800 ± 10	62.0 ± 0.6
#03	7.8 ± 0.3	69.5 ± 0.9	77.3 ± 0.9	911 ± 10	62.9 ± 0.6
#04	7.5 ± 0.2	63.0 ± 0.9	70.5 ± 1.1	1062 ± 30	65.1 ± 0.8
#05	9.2 ± 0.3	101.0 ± 1.1	110.2 ± 1.1	494 ± 10	39.6 ± 0.6
#06	10.2 ± 0.2	67.4 ± 0.8	77.6 ± 0.9	854 ± 15	48.1 ± 0.6
#07	9.9 ± 0.2	60.0 ± 0.5	69.9 ± 0.6	957 ± 12	52.9 ± 0.9
#08	10.3 ± 0.3	52.4 ± 0.8	62.7 ± 1.0	1237 ± 25	60.4 ± 0.9
#09	11.7 ± 0.2	49.3 ± 0.9	61.0 ± 1.0	1299 ± 25	50.8 ± 0.7

a The nanohardness (*H*), Young’s modulus (*E*), elastic energy (*W*_e_), plastic energy (*W*_p_), and total energy (*W*_t_).

The modification of hardness due to doping^13,49^ or alloying^18,50^ was demonstrated in several papers. The PbTe crystal hardening by alloying with CdTe was suggested a long time ago.^51^ According to the current knowledge, crystal hardening could result from several mechanisms. The hardness of an ideal single crystal is proportional to the bond strength and the number of bonds in unit cells in the crystal.^52^ The main hardening mechanism present in the Pb_1–*x*_Cd_*x*_Te solid solution results from the partial replacement of Pb by Cd atoms in the cation sublattice and the a disorder in the crystal lattice. The high dopant–host ionic size mismatch was among the possible reasons discussed in the literature for the hardening of an alloy or doped material.^49^ Adding dopants with a large ionic size mismatch creates local strain, and the strain field may interact strongly with dislocations according to this idea. The result is an enhancement of stress needed for the activation of dislocation propagation, which can finally result in plastic material deformation. No ionic size mismatch effect on the solid solution strengthening was demonstrated recently by the analysis of PbTe with selected impurities.^19^ However, for the analyzed Pb_1–*x*_Cd_*x*_Te solid solutions, this mismatch for Pb and Cd is significantly higher than for all impurities, and for some SSVG crystals the composition is higher than the compositions of materials reported in this paper. Cd is known to increase internal dislocation strain,^53^ so if this effect really exists, it could play some role in our case.

Another proposed solid-solution-related hardening mechanism results from the modification of the valence band (converged band) with an increasing dopant content.^18,50^ This mechanism was not confirmed for PbTe-based materials containing various dopants.^19^ The modification of the valence band for Pb_1–*x*_Cd_*x*_Te solid solution is relatively rapid and the same hole density in the heavy hole ∑ band and the light hole L band was reported for Pb_1–*x*_Cd_*x*_Te SSVG crystals already containing less than 3% CdTe.^26^ One can suppose that the mentioned mechanism could result in a noticeable contribution to Pb_1–*x*_Cd_*x*_Te hardening for a solid solution with a significantly higher composition range than those analyzed in ref (19).

The second type of hardening mechanism is related to Cd point defects. The Cd ions outside the cation sublattice occupy interstitial sites.^42,43^ Highly strained interstitial defects and high vacancy concentrations can result in a significant increase in hardness.^54,55^ This effect was recently observed for Ag- and Cu-doped PbTe.^19^ The interstitial impurity paired with Te vacancies could create a tetragonal distortion in doped PbTe according to the scenario proposed in this paper. Such an asymmetric distortion involving both positive and negative local strain^19^ could be a significant obstacle to the dislocation propagation hampering their motion. The described mechanism should be very effective for Pb1-xCdxTe crystals with a high concentration of Cd interstitials.

The *H* dependences on Cd_XRD_ and Cd_TOT_ are shown in Figure 8. The free carrier concentration dependence of H was reported previously for p-type PbTe- and PbTe-based crystals.^15,19^ The same value of *H* for PbTe SSVG crystal and the MBE layer can be explained by the not very high free carrier concentration in both samples.^26^ Slightly different situations can be expected for Pb_1–*x*_Cd_*x*_Te crystals grown by the two methods due to a higher free carrier concentration in these crystals. A noticeably higher *H* for bulk crystal than for the MBE layer with the same chemical composition of solid solution could probably occur. A qualitatively similar form of *H* increase with increasing Cd content, primarily determined by dominant solid solution hardening mechanisms, should be present for Pb_1–*x*_Cd_*x*_Te bulk crystals and layers with the lattice parameter below that corresponding to the CdTe solubility limit for these layers. In the case of SSVG samples, a typical form of *H* dependence on Cd_XRD_, similar to the square root relation, was observed (Figure 8a).^51,13^ This dependence results primarily from the solid-solution-related hardening mechanisms, and the Cd point-defect-related mechanism is not very significant. For MBE layers, the contribution to *H* related solely to the formation of the solid solution should saturate for samples with a high Cd_XRD_ corresponding to this solution. However, a large *H* scattering was found for some MBE layers containing about 2.1% CdTe in the solid solution. To analyze this finding, the *H* dependence on Cd_TOT_ is plotted in Figure 8b. The present results clearly indicate an increasing role of Cd-related defects outside the cation sublattice and demonstrate important modifications of hardening mechanisms for Cd_TOT_ – Cd_XRD_ > 2% for MBE layers. We believe that the effect of the local lattice distortion resulting from interstitial Cd–Te vacancy complexes, analogous to the effect discussed in ref (19) explains most of the observed strong *H* increase for MBE-grown layers with the highest Cd_TOT_ and these point defects separately correspond to the minor contributions to the crystal hardening.

**Figure 7:**
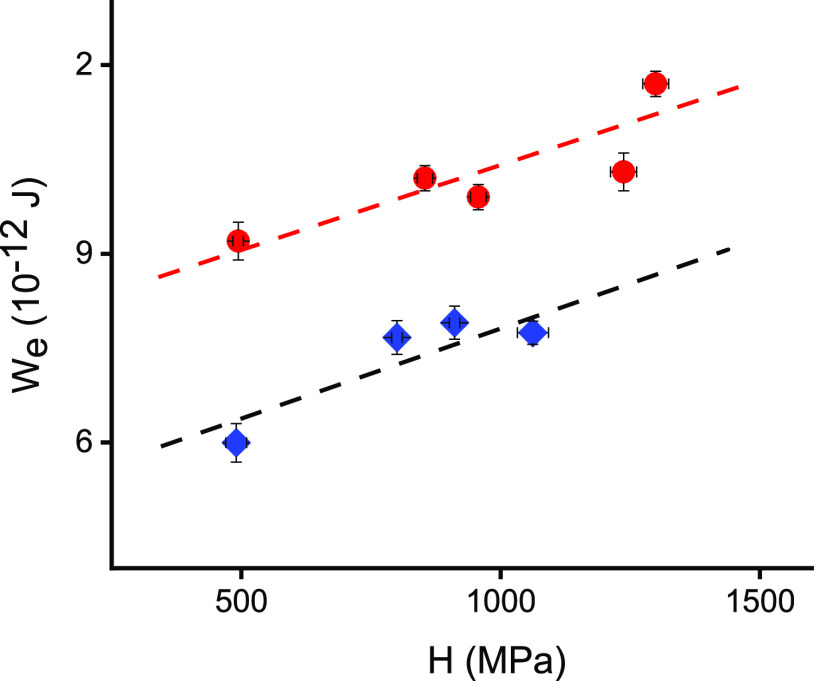
Dependence of the elastic energy (*W*_e_) on the nanohardness (*H*) determined for bulk crystals (blue diamonds) and MBE-grown layers (red circles); dashed lines are the result of the linear fit.

**Figure 8:**
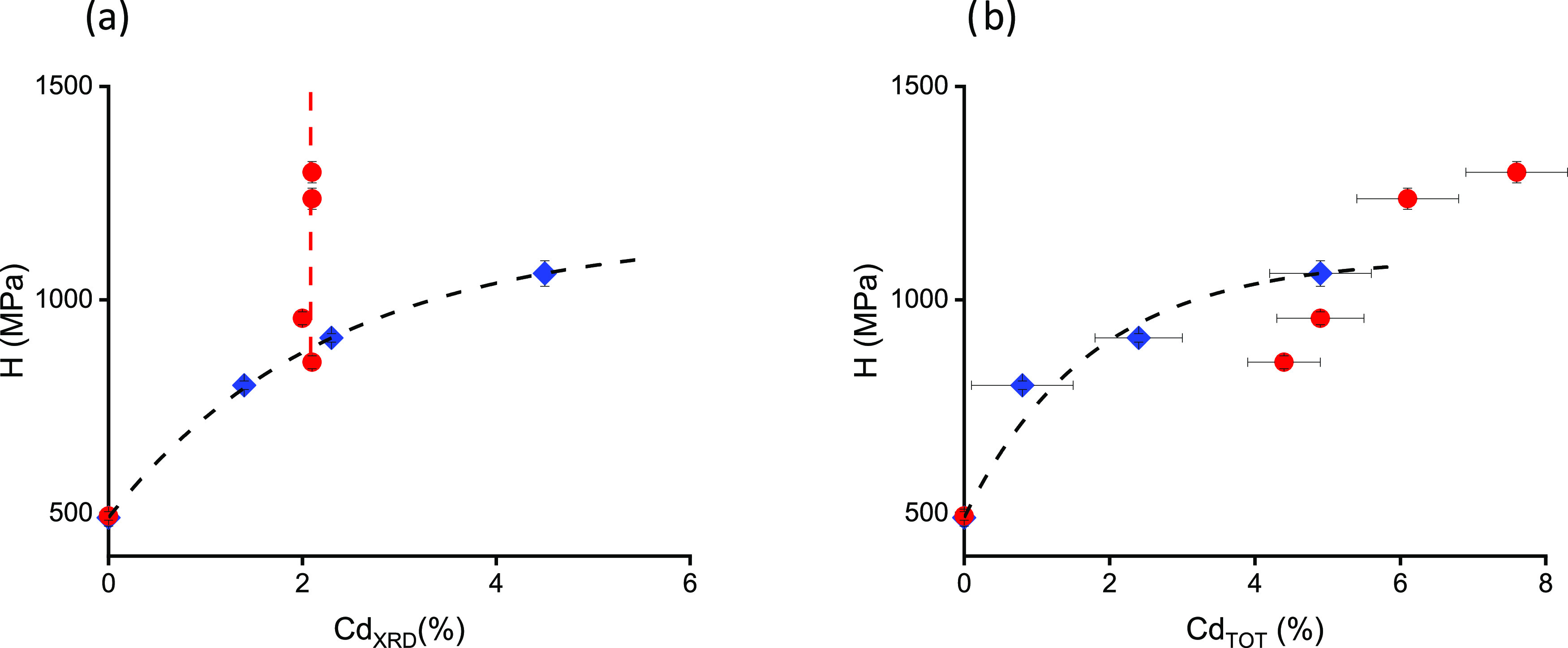
(a) Nanohardness (*H*) versus the CdTe content in the solid solution (Cd_XRD_) for SSVG crystals (blue diamonds) and MBE-grown layers (red circles). The black dashed line guides the eye for bulk crystal data and corresponds to the commonly accepted dependency type; the red dashed line is the result of the linear fit. (b) *H* versus Cd fraction of metal atoms (Cd_TOT_); all symbols are the same as those in (a).

The H results for bulk PbTe crystals are in general agreement with the literature data;^13,56,57^ the nanohardness enhancement of the order of 80% from that of pure PbTe by increasing the Cd content to more than 4% CdTe in the solid solution for SSVG crystals seems to be very high. For comparison, CdTe alloying improved the PbTe nanohardness by about 30% for typical Pb_1–*x*_Cd_*x*_Te ingots containing 3% CdTe.^17^

The bonds in materials with a rock salt structure are arranged in three perpendicular directions, which results in a relatively high hardness anisotropy in comparison to tetrahedrally coordinated compounds.^58^ According to theoretical predictions and available data for other rock salt crystals, the highest hardness is observed along the [001] direction and the lowest one along the [111] direction. The present experimental ratio of *H* along the [001] to *H* along the [111] direction ((1062 ± 30) MPa and (935 ± 10) MPa, respectively) determined for sample #04 is equal to 1.11 and is in excellent agreement with the literature data for PbTe^32^ and Pb_1–*x*_Cd_*x*_Te^31^ irrespective of some difference in H values.

The *E* value determined for PbTe bulk crystal agrees with numerous literature reports^13,19,56,57,59,60^ The present results suggest a slight increase in *E* for SSVG Pb_1–*x*_Cd_*x*_Te crystals with increasing Cd content. A similar effect is observed for MBE layers, but all *E* data determined for these layers are substantially lower. The observed reduction of *E* may be caused by a high concentration of vacancies in these layers. The change in *E* due to vacancies was reported previously for some rock salt metal chalcogenides obtained in the form of composite^61^ or sintered materials.^62^

The measurements of Young’s modulus anisotropy for specimen #04 demonstrated the highest *E* equal to (65.1 ± 0.8) GPa along the [001] direction; this value along the [111] direction was (54.9 ± 1.2) GPa. The present results agree with the trends observed for PbTe and Pb_0.95_Cd_0.05_Te SSVG crystals (ref (32) and ref (31), respectively); the ratio of *E*_001_ to *E*_111_ equal to about 1.19 is much lower than the theoretical *E* anisotropy value for PbS (1.85).^58^ However, for the given direction the *H* and *E* anisotropy experimentally determined by nanoindentation is smaller than the theoretical one. Due to the shape of intender the load applied along the selected direction is partially distributed also along some other directions and the result of measurement corresponds to slightly averaged value.

## Conclusions

A difference in the composition dependence of nanohardness was demonstrated for Pb_1–*x*_Cd_*x*_Te crystals grown by the SSVG and MBE methods. The observed effect was explained by different ratios of hardening mechanisms acting simultaneously in the analyzed materials. It was shown that the hardening observed for SSVG crystals almost fully resulted from the partial replacement of Pb by Cd ions in the cation sublattice. The hardening mechanism resulting from the presence of interstitial Cd defects and possible Cd-related nanoprecipitates, hampering the dislocation propagation, contributed much less to the total hardness.

For MBE layers, the CdTe-in-PbTe solubility limit was equal to 2.1%, and numerous Cd-related point defects were formed with a higher total Cd content. Even for this composition of the solid solution, a further increase in nanohardness with increasing concentration of Cd in investigated layers was shown. These findings pointed out the presence of two types of hardening mechanisms in Pb_1–*x*_Cd_*x*_Te layers: the solid solution-related mechanisms, dominating up to the CdTe-in-PbTe solubility limit for layers, and the Cd point-defect-related hardening mechanism, making an increasing contribution, significant for still higher total Cd contents in these samples. The principal role of the interstitial Cd–Te vacancy complexes was suggested in the latter case.

The Pb_1–*x*_Cd_*x*_Te crystals obtained using different growth methods seem to be a unique system, which can serve as a model system to estimate the role of various hardening mechanisms both in a wide solid solution composition range and for significant modification of two metal atom concentration ratios. Further Pb_1–*x*_Cd_*x*_Te investigations are required to enhance our understanding of the influence of several factors on mechanical properties. In particular, a more precise determination of both types and concentrations of point defects is necessary. To effectively utilize PbTe doped with other selected elements or PbTe-based solid solutions in desired devices, mostly thermoelectric energy converters or optical elements designed for the infrared spectral range, it is necessary to obtain comprehensive knowledge of their mechanical properties and possible failure mechanisms. We believe that our research will be helpful for further studies of the mechanical characteristics of this important group of semiconducting materials.
